# Clinical characteristics and first-line palliative treatment patterns in 3,414 patients with advanced lung cancer in India

**DOI:** 10.3332/ecancer.2025.1867

**Published:** 2025-03-06

**Authors:** Mehak Trikha, Vanita Noronha, Minit Shah, Vijay Patil, Nandini Menon, Ajaykumar Singh, Pratik Chandrani, Omshree Shetty, Rajiv Kumar Kaushal, Trupti Pai, Amit Janu, Nilendu Purandare, Kumar Prabhash

**Affiliations:** 1 Department of Medical Oncology, Tata Memorial Centre, Homi Bhabha National Institute (HBNI), Mumbai 400012, India; 2 Department of Molecular Pathology, Tata Memorial Cent﻿re, Homi Bhabha National Institute (HBNI), Mumbai 400012, India; 3 Department of Pathology, Tata Memorial Centre, Homi Bhabha National Institute (HBNI), Mumbai 400012, India; 4 Department of Radiodiagnosis, Tata Memorial Centre, Homi Bhabha National Institute (HBNI), Mumbai 400012, India; 5 Department of Nuclear Medicine, Tata Memorial Centre, Homi Bhabha National Institute (HBNI), Mumbai 400012, India; † Have contributed equally for this work; a https://orcid.org/0000-0001-8858-5004

**Keywords:** advanced lung cancer, genomic profile, palliative, targeted therapy, epidemiology trend

## Abstract

Lung cancer, particularly non-small-cell lung cancer (NSCLC), is a major global health issue. In India, the complexity of managing this disease is heightened by diverse demographics, varying healthcare access and evolving epidemiological trends influenced by factors such as smoking and advancements in diagnostics. This study explores cancer demographics and first-line palliative treatment options. We conducted a retrospective analysis of 3,414 advanced lung cancer patients planned for palliative systemic therapy at our centre between March 2000 and June 2017. The mean age of the cohort was 56.7 interquartile range (IQR: 21–88) years with a male predominance (71.9%). Histological subtypes included adenocarcinoma (82.9%), squamous cell carcinoma (13.6%) and others (3.5%). Intrathoracic metastases were seen in 40.7% and intrathoracic and extrathoracic in 37.7% of patients. The extrathoracic sites were skeletal (32.5%), liver (14.4%), brain (13.7%) and adrenal gland (9.0%). The baseline Eastern Cooperative Oncology Group Performance status was 0 (8.3%), 1 (58.7%), 2 (20.7%), 3 (11.7%) and 4 in 0.6% of the patients. Epidermal growth factor receptor (EGFR) and anaplastic lymphoma kinase (ALK) positivity rates were lower in smokers. Most patients (92.4%) received the first-line systemic therapy, predominantly platinum-based doublets (67.1%). Tyrosine kinase inhibitors were given to 51.8% of EGFR+, 48% of ALK+ and 54.1% of mutation-negative cases. This study provides crucial insights into lung cancer demographics and treatment patterns at a single tertiary care centre in India, highlighting an increase in female lung cancer cases, steady smoking rates and improved access to genetic testing and targeted therapies.

## Introduction

Lung cancer is one of the most common malignancies globally, contributing to approximately 2.2 million new cases and 1.8 million deaths as of 2020 [1]. The heterogeneity of lung cancers is evident in histological and molecular aspects [2]. Histologically, they are divided into non-small-cell lung cancer (NSCLC, making up 85% of cases) and small-cell lung carcinoma (constituting 15% of cases) [2, 3]. NSCLCs are further classified histologically into adenocarcinoma (ADC), squamous cell carcinoma (SCC) and large-cell carcinoma [2]. Cigarette smoking is the primary cause of lung cancer, followed by additional risk factors such as prolonged exposure to air pollution, occupational carcinogenic exposures and genetic predisposition [4, 5]. Notably, lung cancer incidence in never-smokers and younger individuals is often linked with the ADC subtype’s higher propensity of driver mutation [6].

Most patients with NSCLC present with advanced disease [7, 8]. Traditionally, chemotherapy was the base for the treatment of advanced lung cancer. However, identifying oncogenic driver mutations in NSCLC has revolutionised therapeutic strategies, improving survival outcomes [9, 10]. Genomic studies have identified driver mutations associated with primary lung cancer, including epidermal growth factor receptor (EGFR) [11, 12], anaplastic lymphoma kinase (ALK) [13], ROS1 Proto-Oncogene Tyrosine Kinase Receptor (ROS1) [14] and Serine/Threonine-Protein Kinase BRAF (BRAF) [15]. The advent of targeted therapy drugs has increased the survival rates from a median overall survival of 11 months to a 5-year survival rate of 17.8% [16]. However, challenges persist, particularly in low- and middle-income countries (LMICs), where access to molecular testing and targeted therapy remains limited. In countries like India, molecular diagnostic testing facilities are primarily concentrated in referral hospitals, academic centres or large private laboratories, leading to delayed molecularly profiled cancer diagnoses [8, 17].

Despite being one of the most common cancers, there are limited data on its demography, epidemiology trends and treatment patterns. This study explores the clinical profile, epidemiological trends, access to molecular testing and targeted therapy from a tertiary centre in India.

## Material and methods

We performed a retrospective analysis of patients presenting with advanced lung cancer planned for palliative intent systemic therapy at our centre between March 2000 and June 2017. We included the data of patients presenting to the outpatient or in-patient department of the Thoracic Medical Oncology Department with a histologically confirmed diagnosis of lung cancer. They were the patients who were part of the prospectively maintained database and patients willing to participate and provided consent for the same. The management of all patients was based on a multidisciplinary board decision. Data of patients treated with a curative intent were excluded from the analysis.

Patient data were extracted from the Electronic Medical Records (EMR) and the patient’s case file. Patients were telephonically contacted if follow-up data were unavailable in the EMR/case file. The data collected included the patient’s demographic profile, which included age, sex, performance status, comorbidities, smoking habit, tumour characteristics, tumour pathology, site of metastasis, mutation subtype and the first line of treatment received. Patients with NSCLC histology underwent molecular testing with either reverse transcription-polymerase chain reaction (RT-PCR) for EGFR, immunohistochemistry (IHC) or fluorescence *in-situ* hybridisation (FISH) ALK and ROS1 mutation and next-generation sequencing (NGS) based on feasibility. The study was conducted according to the ethical principles of the Declaration of Helsinki and the Indian Council of Medical Research guidelines. Informed consent was not required due to its retrospective design. The Institutional Ethics Committee has assigned trial number 4,310 for our study. The IEC register number of the trial is 4,310. Informed consent waiver was approved as it is a retrospective study.

### Aim and objectives

To explore the clinical characteristics, demographic profile and treatment patterns in advanced non–small-cell lung cancer patients at a tertiary cancer centre in India and to study the change in epidemiological trends, genomic profile and treatment patterns from 2000 to 2017.

### Statistics

A formal sample size calculation was not done as this was a retrospective analysis. The data analysis was performed using the Statistical Package for the Social Sciences software (IBM SPSS Statistics for Windows, Version 25.0. IBM Corp., Armonk, NY, USA). Descriptive statistics were used for baseline demographic details using absolute numbers, simple percentages, median, range and IQR. Differences between patient groups were tested using the chi-squared or Fisher’s exact test. In all analyses, *p*-value <0.05 indicated statistical significance.

## Results

### Baseline characteristics

This study included a cohort of 3,414 patients with a mean age of 56.7 (IQR: 21–88) years and a male predominance (*n* = 2,455, 71.9%). In this cohort, 1,765 (51.7%) patients were smokers. ADC was the most common histological subtype seen in 2,830 (82.9%) patients, followed by SCC in 464 (13.6%) and others in 120 (3.5%) patients. The baseline Eastern Cooperative Oncology Group Performance Status (ECOG-PS) was 0 in 284 (8.3%), 1 in 2,004 (58.4%), 2 in 706 (20.7%), 3 in 400 (11.7%) and 4 in 20 (0.6%) patients. The most common sites of metastasis were intrathoracic in 1,141 (40.7%) and intrathoracic and extrathoracic in 1,288 (37.2%) patients. The most common extrathoracic areas were the skeletal (32.5%), followed by the liver (14.4%), brain (13.7%) and adrenal gland (9.0%) (Table 1).

Of the 3,294 patients with NSCLC, driver mutation testing of EGFR by RT-PCR alone or as part of NGS was performed in 2,394 (72.6%) patients. EGFR mutations were observed in 607 (24.1%) patients (Table 2). ALK mutation testing by FISH/IHC was done in 2,200 (66.7%) patients, of whom 272 (12.4%) were positive.

## Epidemiological trend

**Figure 1.**
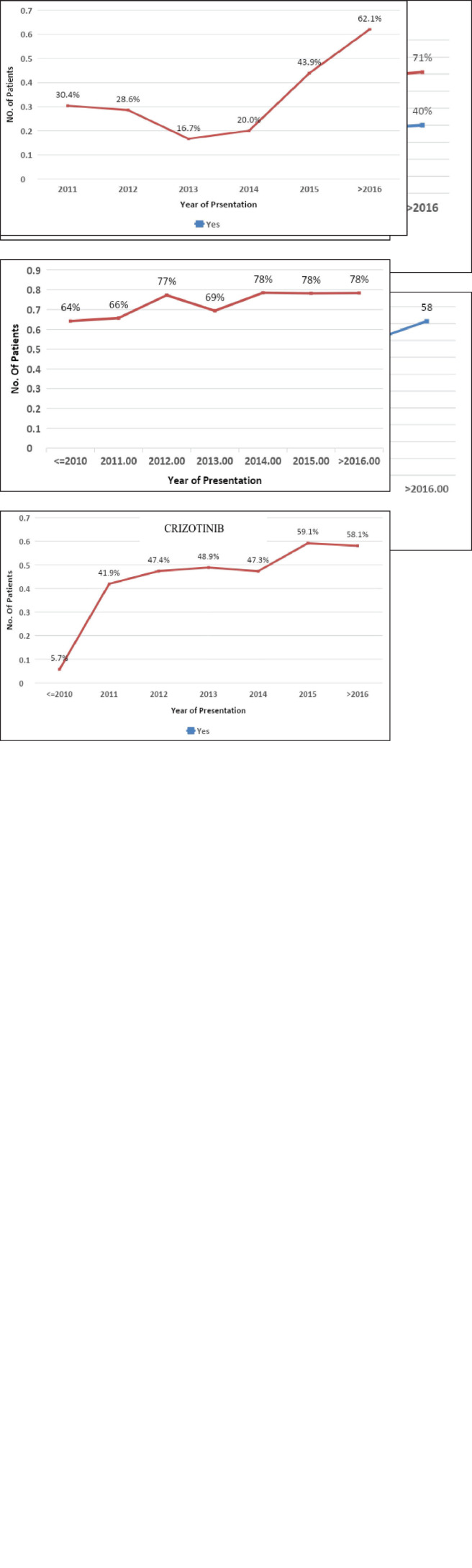
Epidemiological trends. (i): Epidemiology trend in gender (female – 28.4% in <2010 to 40.2% in >2016). (ii): Epidemiological trend in age (stable mean age – 57 years). (iii): Epidemiological trends in histology (ADC 68.6% higher incidence compared to SCC through the study period). (iv): Epidemiological trend in genomic testing (68.1% in 2010 to 78.2% at >2016). (v): Epidemiological trend in use of EGFR TKIs (5.7% in <2010 to 58.1 in >2016). (vi): Epidemiological trend in use of crizotinib (30.4% in 2011 to 62.10% at >2016).

Throughout the study period, the mean age of patients diagnosed with lung cancer was consistent (mean age: 56 years). There was an increase in the proportion of female patients with lung cancer, rising from 28.4% in 2010 to 40.2% in >2016, while the male distribution remained constant. The percentage of smokers declined from 59.4% in 2010 to 44.2% in 2016. Notably, ADC histology showcased a substantial 69.8% increase compared to SCC over the entire study duration. The utilization of molecular testing for driver mutations via RTPCR/IHC/NGS witnessed a rise from 68.1% in 2010 to 78.2% in the period >2016 (Figure 1i–iv).

Comparing smokers with nonsmokers, we observed a higher smoking incidence in the >60-year-old age group (42.7% versus 34.5%) with a marked male predominance (92.3%). Smokers displayed a greater incidence of SCC (19.7% versus 6.6%), although ADC histology was more prevalent in both groups. Smokers exhibited significantly lower EGFR and ALK positivity rates (11.8% versus 24.5% and 5.3% versus 10.7%, respectively) (Table 3).

## Treatment pattern

First-line systemic therapy was initiated in 3,256 (92.4%) patients. The most common chemotherapy was platinum-based doublet in 2,118 (67.1%) patients. The most common regimen was pemetrexed platinum in 1,471 (46.6%) patients. Tyrosine kinase inhibitors (TKIs) were used as the first-line treatment in 973 (29.5%) NSCLC patients. The 319 (51.8%) EGFR-positive and 127 (48%) ALK+ patients received first-line TKIs. Their usage increased throughout the study. EGFR inhibitor usage in EGFR-positive patients increased from 5.7% to 58.1%, and ALK inhibitor usage in EGFR-positive patients increased from 30.2% in 2011 to 62.1% at >2016. The 527 (54.1%) patients received TKI on compassionate grounds in patients with a driver mutation-negative status (Figure 1v and vi). Four patients with brain metastases underwent surgery, 35 (7.4%) underwent stereotactic radiosurgery and 432 (91.7%) underwent whole brain radiotherapy.

## Discussion

To our understanding, this study represents the most extensive single-centre experience examining the demographic profile, treatment patterns and epidemiological trends in the first-line management of patients with advanced non–small-cell lung cancer. The mean age of our cohort was 56 years, consistent with earlier studies conducted in India [18–22]. Yet, intriguingly, it diverges from the age groups reported in Western literature, which is a decade higher [19–25]. The higher number of male lung cancer patients in our study aligns with the Indian population but exceeds the male dominance seen in Western studies. This divergence can be attributed to the higher prevalence of smoking among Western women, which is a well-established risk factor for NSCLC. The prevalence of smoking in our cohort (51.7%) was in agreement with a previous study by Noronha *et al* [6, 35] but significantly lower than the rates observed in Western countries (80%–90%) [23, 24, 26]. The stability in smoking trends throughout the study period suggests the involvement of other contributory factors in lung cancer aetiology, such as passive smoking, air pollution and indoor smoke exposure, which are more prevalent in the rural regions of India [27, 28]. Throughout the study period, the incidence of ADC histology prevailed over SCC, reflecting the evolving global patterns of NSCLC. This shift can be attributed to the changing dynamics of smoking habits, an increasing incidence of lung cancer among women and improved accuracy in histopathological reporting.

The study revealed a significantly higher prevalence of smoking in the older population (>60 years old), with 42.7% of patients being smokers, in contrast to 34% of nonsmokers. This observation aligns with established trends, suggesting that there is a higher likelihood of smoking in older patients [6]. In addition, the high prevalence of male smokers (92.3%) highlights the well-known gender gap in smoking habits, where men typically smoke more than women [6]. One notable finding was the discrepancy in histological subtypes between smokers and nonsmokers. Smokers exhibited a substantially higher percentage of SCC, with 19.7% of smokers having SCC compared with 6.6% of nonsmokers. This finding was similar to that found in the literature for SCC [19]. Conversely, ADC was the most prevalent histology in both groups. This study also revealed differences in the molecular profiles of smokers and nonsmokers with NSCLC. Smokers exhibited significantly lower rates of EGFR and ALK positivity, with 11.8% of smokers having EGFR mutations compared with 24.5% of nonsmokers and 5.3% of smokers with ALK mutations compared with 10.7% of nonsmokers. These findings reflect the evolving understanding of the impact of smoking on molecular alterations in NSCLC. EGFR, which has been strongly associated with nonsmoking status, particularly in Asian populations [20], has a lower EGFR mutation rate among smokers, which concurs with this known pattern, suggesting that other factors, such as smoking-related mutational burden, may influence the genetic makeup of NSCLC in smokers. Conversely, ALK rearrangements have been associated with younger age and nonsmoking status [21]. The lower prevalence of ALK mutations among smokers could be attributed to distinct mutational profiles associated with smoking-related carcinogenesis.

The proportion of patients with good performance status (ECOG 0–1) at the time of presentation (67.0%) and those with poor performance status (ECOG 2–4, 33.0%) was comparable to that of the Western population [29]. This shift indicates an evolving healthcare landscape and emphasises the need for timely diagnosis and intervention. The study demonstrated a genetic testing rate of 76.3%, revealing a consistent increase from 64% in 2010 to 78% in 2016, mirroring trends observed in Western populations [30]. The introduction of EGFR testing by RT-PCR in 2005, ALK testing by FISH in 2011 and NGS in 2019 reflects the progressive integration of advanced molecular diagnostics at the study centre. Among the tested cohorts, the EGFR positivity rate of 24.1% aligns with findings from Indian studies but surpasses that of Western populations [31–37]. In contrast, the ALK positivity rate of 12.4% exceeds that of the Western population (5%) and ranges from 1.5% to 7.6% in Indian individuals [38–40]. However, it is crucial to acknowledge that testing for ALK mutations was not uniform throughout the study period, which may have affected the observed rate. Moreover, other mutations (mesenchymal–epithelial transition factor) and ROS were underrepresented due to comprehensive panel testing initiated only in EGFR/ALK-negative patients from 2015 onwards. Regarding treatment, 92.4% of the patients initiated first-line systemic therapy, including chemotherapy and targeted therapy reflecting high treatment rates. However, the inability of 158 (4.6%) patients to commence therapy due to poor performance status (1.7%) or logistic challenges (2.9%) highlights the need for targeted interventions to address these barriers, particularly logistical support.

One hundred fifty-eight (4.6%) patients did not receive first-line systemic therapy at our centre; 59 (1.7%) were due to poor performance status and 99 (2.9%) were due to logistic reasons.

Notably, 51.8% of EGFR-positive patients were exposed to TKIs as first-line treatment, albeit lower than the reported real-world data from the USA (72.8%) during a similar period [41]. For ALK-positive patients, 48% received Crizotinib as first-line therapy, aligning with the American literature and exceeding the rates reported in a European study [42, 43]. The increased utilization of Crizotinib in our population is attributed to the amplified support from various governmental and nongovernmental organizations in terms of providing accessibility to the drug.

Over the study period, the increased EGFR and ALK inhibitor usage underscores evolving strategies to enhance access to targeted therapy, including government and private initiatives. Over 50% of patients were administered TKIs on compassionate grounds, considering their compromised baseline performance status or a strong suspicion of mutation positivity based on medical history. Survival advantage in this group of patients has been shown in a study by Shah *et al* [44]. This opens doors for LMICs with limited resources. In addition, although 3,256 (92.4%) patients commenced first-line systemic therapy, 73 (2.2%) patients were lost to follow-up after initiation, prior to response evaluation. Consequently, the extent of therapy administered to these individuals remains uncertain. This illustrates the challenges associated with ensuring continuity of care, which may adversely affect outcomes, and highlights the necessity for robust patient tracking mechanisms. Furthermore, this attrition underscores the critical need to address nonclinical determinants such as accessibility and adherence to follow-up to enhance treatment delivery and improve outcomes.

## Limitations

A survival study could not be conducted due to a significant loss of follow-up data due to logistical and financial constraints.

## Conclusion

This study contributes significantly to the comprehensive understanding of demographic profiles, epidemiological trends and access to targeted therapy within a single centre. Our centre’s trajectory appears to align with global histopathological trends in lung cancer. Notably, there was an increase in the proportion of female lung cancer cases, whereas rates of smoking and mean age at diagnosis exhibited no significant changes over time. The expansion of the healthcare sector is evident with the increased use of genetic testing and enhanced patient access to targeted therapies. However, additional efforts are required to address barriers to continuity of care, including follow-up adherence and accessibility, to optimise treatment outcomes in lung cancer.

## Conflicts of interest

None.

## Funding

None.

## Institutional review

The study was conducted according to the ethical principles of the Declaration of Helsinki and the Indian Council of Medical Research (ICMR) guidelines. Informed consent was not required due to its retrospective design and waiver for the same was approved by the ethics committee. The Institutional Ethics Committee (IEC) of Tata Memorial Hospital approved the study.

## Author contributions

Mehak Trikha: Conceptualization, methodology, resources, and project administration; writing - original draft preparation and writing: review and editing.

Vanita Noronha: Methodology and investigation; data curation; formal analysis; writing - original draft preparation and writing - Review and Editing.

Minit Shah: Resources, project administration and writing - review and editing.

Vijay Patil: Data curation, methodology and supervision.

Nandini Menon: Data curation and writing: review and editing.

Ajaykumar Singh: Data curation and writing - review and editing.

Prateek Chandrani: Data curation and resources.

Omshree Shetty: Resources, project administration and supervision.

Rajiv Kumar: Resources, project administration and supervision.

Trupti Pai: Resources and writing: review and editing.

Amit Janu: Supervision and resources.

Nilendu Purandare: Resources and writing: review and editing.

Kumar Prabhash: Writing: review and editing; conceptualization, methodology, project administration and supervision.

## Data availability statement

All data underlying the results are available as a part of the article, and no additional source data are required.

## Figures and Tables

**Table 1.** Baseline characteristics.

Baseline characteristics	*N* = 3,414
Age	56.7 (IQR:21–88)
Gender	
Male	2,455 (71.9%)
Female	959 (28.1%)
Smoker	1,765 (51.7%)
Co-morbidities	
Diabetes mellitus-2	570 (16.7%)
Hypertension	800 (23.4%)
IHD	94 (2.8%)
COPD	188 (5.5%)
ECOG-PS	
0	284 (8.3%)
1	2,004 (58.7%)
2	706 (20.7%)
3	400 (11.7%)
4	20 (0.6%)
Stage	
III	610 (17.9%)
IV	2,804 (82.1%)
Histology	
ADC	2,830 (82.9%)
SCC	464 (13.6%)
Others	120 (3.5%)
Site of metastasis (*N* = 2,800)	
Intrathoracic-lung	419 (14.9%)
Intrathoracic-pleura	722 (21.1%)
Both	1,288 (37.7%)
Skeletal	1,111 (32.5%)
Adrenal	308 (9.0%)
Liver	492 (14.4%)
Brain	471 (13.7%)
Leptomeningeal	20 (0.6%)

IQR – Interquartile range, IHD – Ischemic heart disease, COPD – Chronic Obstructive Pulmonary Disease, ECOG-PS – Eastern Cooperative Oncology Group – Performance status, ADC – Adenocarcinoma, SCC – Squamous cell carcinoma

**Table 2.** Mutation analysis.

Mutational analysis	(*N* = 3,294)
Not done	900 (27.3%)
Negative (*N* = 2,515)	1,623 (64.5%)
EGFR mutation	615 (24.11%)
EGFR-EXON 19	358 (58.9%)
EGFR–EXON 21	223 (36.7%)
EGFR–EXON 18	14 (2.3%)
EGFR-EXON 20	12 (1.9)
ALK mutation (*N* = 2,200)	272 (12.4%)

EGFR – Epidermal Growth Factor Receptor, ALK – Anaplastic Lymphoma Kinase

**Table 3.** Comparison of characteristics between smokers and nonsmokers with lung cancer.

Characteristics	Smokers (*N* = 1,765)	Nonsmokers (*N* = 1,649)	*p*-value
Age (years)			
<40	85 (4.8%)	156 (9.4%)	
40–60	926 (52.5%)	923 (56.1%)	
>60	754 (42.7%)	570 (34.5%)	*p* = 0.00
Gender			
Male	1,629 (92.2%)	823 (49.9%)	
Female	136 (7.8%)	826 (50.1%)	
Histology			
ADC	1,337 (75.7%)	1,493 (90.5%)	
SCC	355 (20.1%)	109 (6.7%)	*p* = 0.00
ECOG-PS			
0–1	1,001 (56.7%)	1,287 (78.1%)	
2–4	764 (43.3%)	362 (21.9%)	
Mutation			
EGFR	210 (11.8%)	405 (24.5%)	*p* = 0.00
ALK	95 (5.2%)	177 (10.7%)	*p* = 0.00
Treatment			
Yes	1,620 (91.8%)	1,547 (93.8%)	*p* = 0.02
No	145 (8.2%)	102 (6.2%)	

ECOG-PS – Eastern Cooperative Oncology Group – Performance status, ADC – Adenocarcinoma, SCC – Squamous cell carcinoma, EGFR – Epidermal Growth Factor Receptor, ALK – Anaplastic Lymphoma Kinase
